# Supplementary material for: Vitamin D Modified DSS-Induced Colitis in Mice via STING Signaling Pathway
Source: Biology (Basel). 2025 Jun 18;14(6):715. doi: 10.3390/biology14060715 (PMC12190092; doi:10.3390/biology14060715)
Supplement: Supplementary file 1 [file biology-14-00715-s001.zip › Supplementary Materials S1: 16s-report of gut microbiota/Groups/diff_analysis/metagenomeRCAZY/figure/boxplot/alllog.pdf]

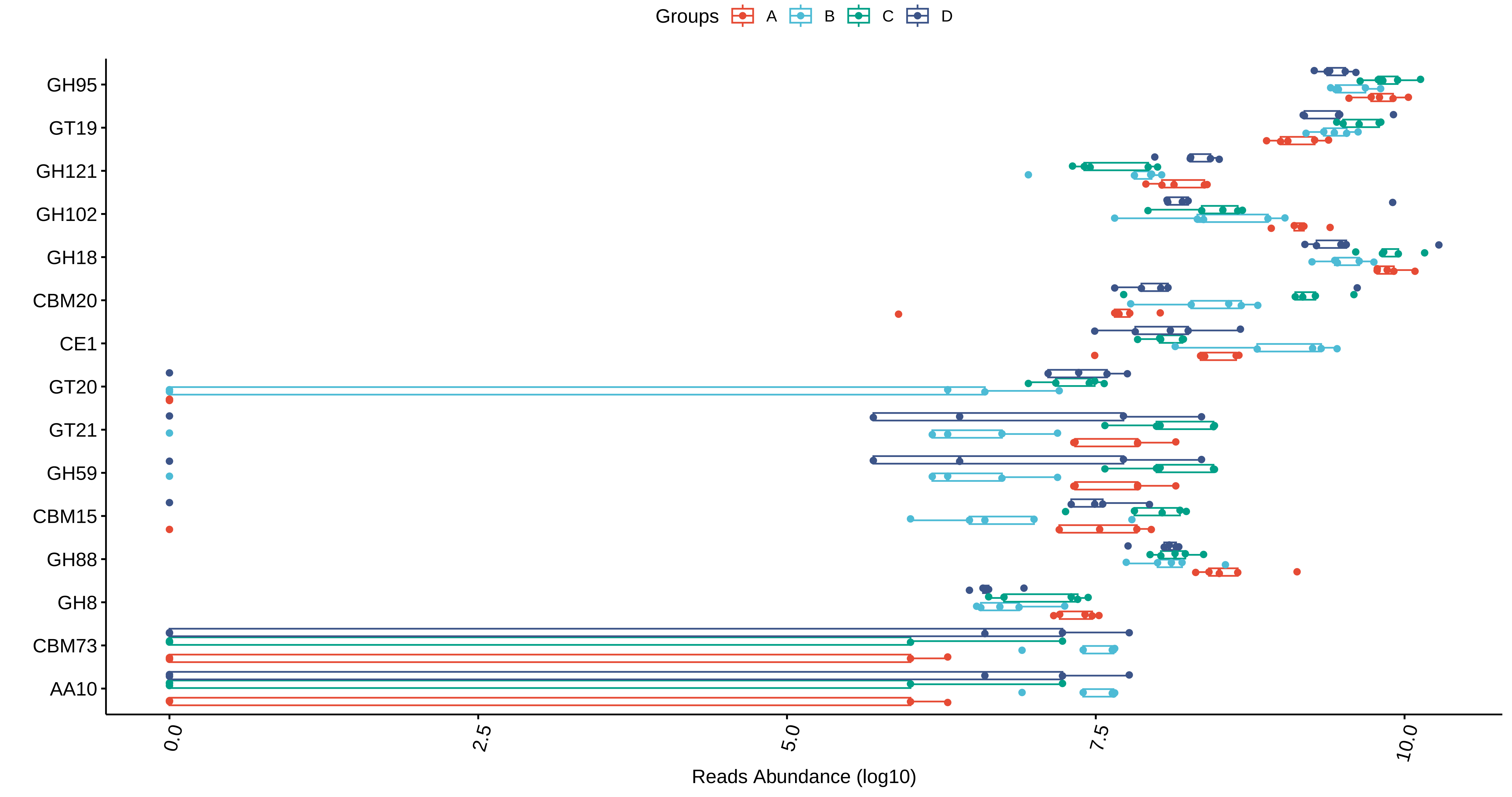

Groups
A
B
C
D
GH95
GT19
GH121
GH102
GH18
CBM20
CE1
GT20
GT21
GH59
CBM15
GH88
GH8
CBM73
AA10
0.0
2.5
5.0
7.5
10.0
Reads Abundance (log10)
